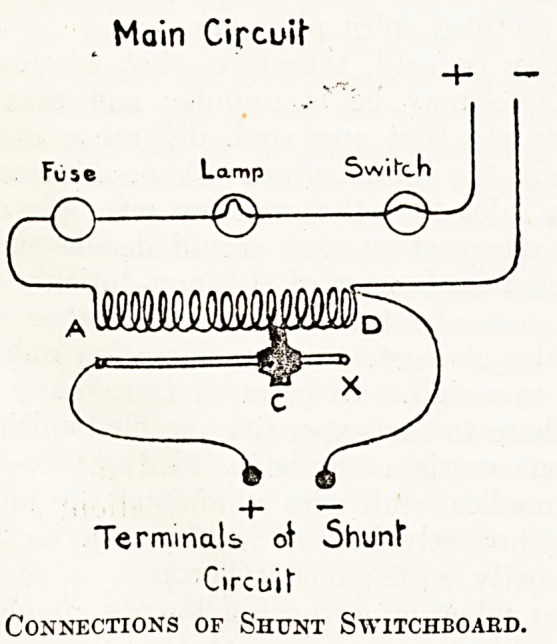# The Use of Shunt Rheostats for Hydro-Electric Baths

**Published:** 1912-11-16

**Authors:** 


### THE DANGERS TO LIFE OF ELECTRIC BATHING.

### The Use of Shunt Rheostats for Hydro-Electric Baths.

Surely the danger of giving hydro-electric baths from a shunt rheostat has already been sufficiently emphasised to impress even the most ignorant. That the practice still obtains, however, in certain establishments has once more been brought home to

Connections of Shunt Switchboard.

the general public by the recent sad death of a professional man who was literally electrocuted in an electric bath in the West End of London. The dangers of an accident with a shunt rheostat are twofold: The first, and most likely to happen—viz. an *earth shock*—was explained at some length in Dr. Norman's article in The Hospital for March 9; the second, that of *short-circuit*, will be considered in an article on hydro-electric baths shortly to appear in this journal. The present fatality was caused by an accident of the latter kind. Evidently the filament of the resistance lamp broke, the two pieces falling across each other in such a way that all the resistance of the lamp was short-circuited, the result being that the patient received a fatal amount of current through his body. Thus we see that in using this type of resistance the patient's life, to quote an apt phrase from the *Standard*, "literally hangs by a thread—the brittle filament of an electric lamp."

A glance at the diagram, which was published in The Hospital for March 9, 1912, which we here reproduce, will explain how such an accident may happen. Two-thirds of the total resistance in the main circuit of the rheostat is situated in the lamp filament, and if this is removed (by *short-circuit*) the voltage in the shunt circuit at once rises to almost that of the street mains, hence it forces a fatal amperage through the patient's body. By taking certain precautions the risk of *earth shock* can be obviated, and by using four resistance lamps (in series) the danger from short-circuit can be considerably diminished, but even then the leading wires to the lamps may become short-circuited by some accident; hence we are forced to the conclusion that a shunt rheostat for hydro-electric baths is unjustifiable, if not actually criminal.

## Figures and Tables

**Figure f1:**